# The complete mitochondrial genome of *Cheilinus trilobatus* (Perciformes: Labridae)

**DOI:** 10.1080/23802359.2022.2161835

**Published:** 2023-01-02

**Authors:** Teng Wang, Yupei Li, Qin Ma, Yong Liu, Yayuan Xiao, Peng Wu, Lin Lin, Chunhou Li

**Affiliations:** a Key Laboratory of South China Sea Fishery Resources Exploitation & Utilization, Ministry of Agriculture and Rural Affairs, Guangdong Provincial Key Laboratory of Fishery Ecology and Environment, South China Sea Fisheries Research Institute, Chinese Academy of Fishery Sciences, Guangzhou, China; b Southern Marine Science and Engineering Guangdong Laboratory (Guangzhou), Guangzhou, China; c Observation and Research Station of Pearl River Estuary Ecosystem, Guangzhou, China; d Sansha Marine Protected Area Administration, Sansha, China; e College of Life Science, Nanchang Normal University, Nanchang, China

**Keywords:** *Cheilinus trilobatus*, mitochondrial genome, phylogenetic analysis

## Abstract

*Cheilinus trilobatus* Lacépède, 1801 is a species of genus *Cheilinus*. In this study, we sequenced the complete mitochondrion genome of *C. trilobatus*. The mitochondrial genome was 17,292 bp, consisting of 13 protein-coding genes, 22 tRNA genes, two rRNA genes, and one non-coding control region (D-loop). The nucleotide composition was 27.31% A, 25.1% T, 17.23% G, and 30.36% C. Phylogenetic analysis suggested that *C. trilobatus* was closely related to *Cheilinus oxycephalus*. The complete mitogenome of *C. trilobatus* provided basic data for the genetic diversity conservation of this species.

## 1. Introduction

*Cheilinus trilobatus* Lacépède, 1801 is a common species belonging to the family Labridae. It usually inhabits in shallow reefs, especially in areas with rich coral and algal cover (Khalaf and Disi 1997). It feeds on mollusks, crustaceans, and sea urchins, and occasionally takes fishes (Myers 1991). Previous studies on this species have focused on its chromosome analysis and levels of heavy metals. However, the study about the molecular biological properties of *C. trilobatus* has not been published. In this study, we first sequenced the complete mitochondrial genome of *C. trilobatus*, and performed a phylogenetic analysis among *Cheilinus* with the available mitogenomic sequences. It may provide some basic data for the genetic diversity conservation of this species.

## 2. Materials

**Figure 1.**
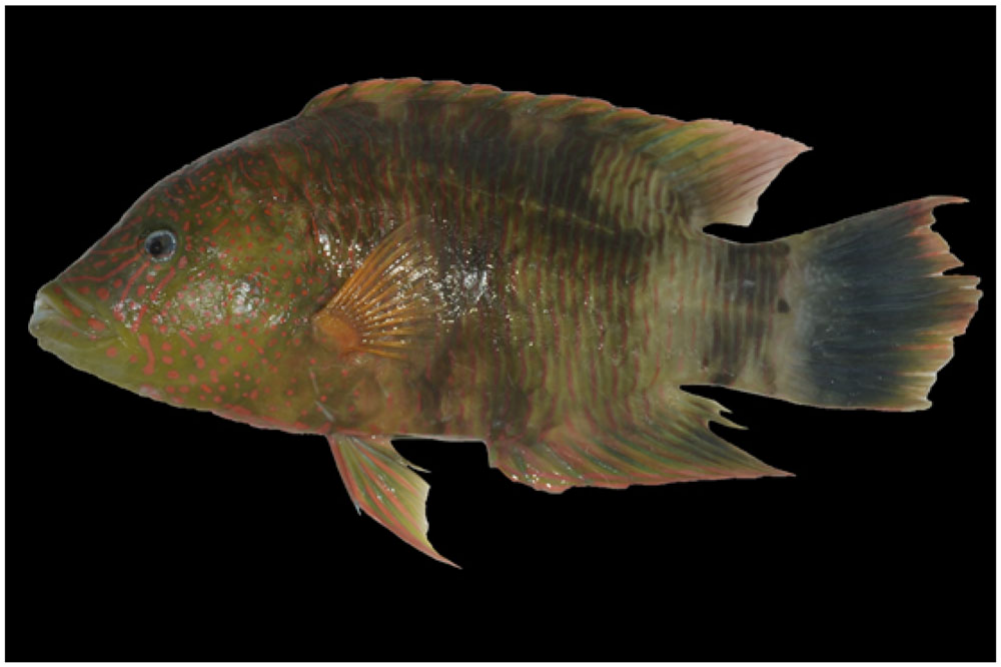
Specimen of *Cheilinus trilobatus.* This species only has one dorsal fin and the third fin spine is the longest dorsal spines. The photo was taken by Teng Wang.

*Cheilinus trilobatus* was obtained from the Qilianyu (16°55′–17°00′N, 112°12′–112°21′E), Xisha Islands, China in April 2020 (Figure 1). All specimens were deposited at South China Sea Fisheries Research Institute, Chinese Academy of Fishery Sciences (wt3074589@163.com, Voucher specimen: SYCY20210420001).

## 3. Methods

Genomic DNA was extracted from an adult’s dorsal muscle using E.Z.N.A.^®^ Tissue DNA Kit (OMEGA, China) following the manufacturer’s instructions. DNA library preparation and 150-bp paired-end sequencing were performed on the Illumina HiSeq platform. Raw reads used for assembling the mitochondrial genome were deposited into the NCBI SRA (SRA accession: SRR18531815). The raw data was assembled by using GetOrganelle version 1.7.5.3 (Jin et al. 2020). The complete mitochondrial of *C. trilobatus* was annotated by MITOS2 web server (Bernt et al. 2013). Protein-coding genes (PCGs) and rRNAs were annotated by comparing with the complete mitochondrial genome of the *Cheilinus undulatus* Rüppell, 1835 (Matthew et al. 2018). The 13 common protein-coding genes in each complete mitochondrial genome of 12 species were aligned with the genes in *C. trilobatus* using MAFFT 7.037 (Katoh and Standley 2016). Then, model-finder var 1.6 was run to select the best-fit model and the mtVer + F + R3 model was chosen. Finally, iqtree 2.0 was used to construct a phylogenetic tree with 1,000 bootstraps based on the ML method.

## 4. Results

**Figure 2.**
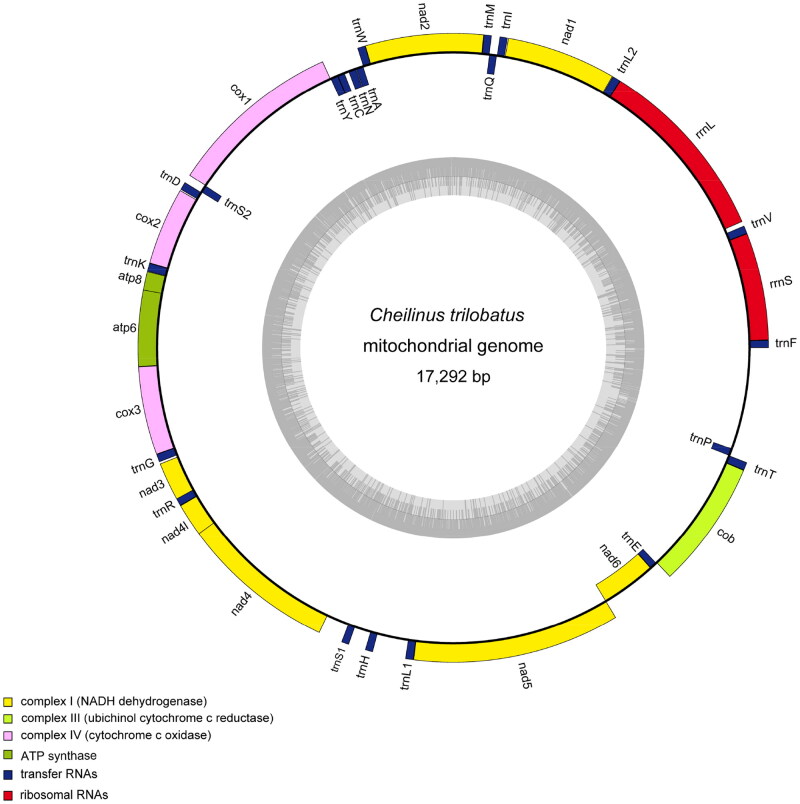
Mitochondrial genome map of *Cheilinus trilobatus.* The circular map of *C. trilobatus* was drawn using the OGDRAW program. The map consists of two circles and information about each circle is as follows: the inner circle indicates the GC content, and the external circle indicates the genes having different colors based on their functions.

The annotated complete mitochondrial genome sequence was submitted to NCBI (GenBank: OM994969). It was 17,292 bp in length containing 13 PCGs (*Cyt* b, *ATP6*, *ATP8*, *COX1*-*3*, *ND1*-*6*, *ND4L*), 22 tRNA genes, two rRNA genes (12S and 16S rRNA), and one control region (CR or D-Loop) (Figure 2). Eight tRNA genes (*Gln*, *Ala*, *Asn*, *Cys*, *Tyr*, *Ser*, *Glu*, and *Pro*) and NADH dehydrogenase subunit 6 (*ND6*) are encoded on the light strand (L-strand), the other 29 genes are encoded on the heavy strand (H-strand). The nucleotide composition was 27.31% A, 25.1% T, 17.23% G, and 30.36% C. Almost all of 13 PCGs for *C. trilobatus* share the regular initiation codon ATG except *COI* gene with GTG, and *ND3* gene with GTT. There are three different patterns of termination codons: eight PCGs terminated with the stop codons TAA or TAG, whereas five PCGs (*COX2*, *COX3*, *ND3*, *ND4*, *Cyt b*) end with incomplete form TA − or T–.

## 5. Discussion and conclusion

**Figure 3.**
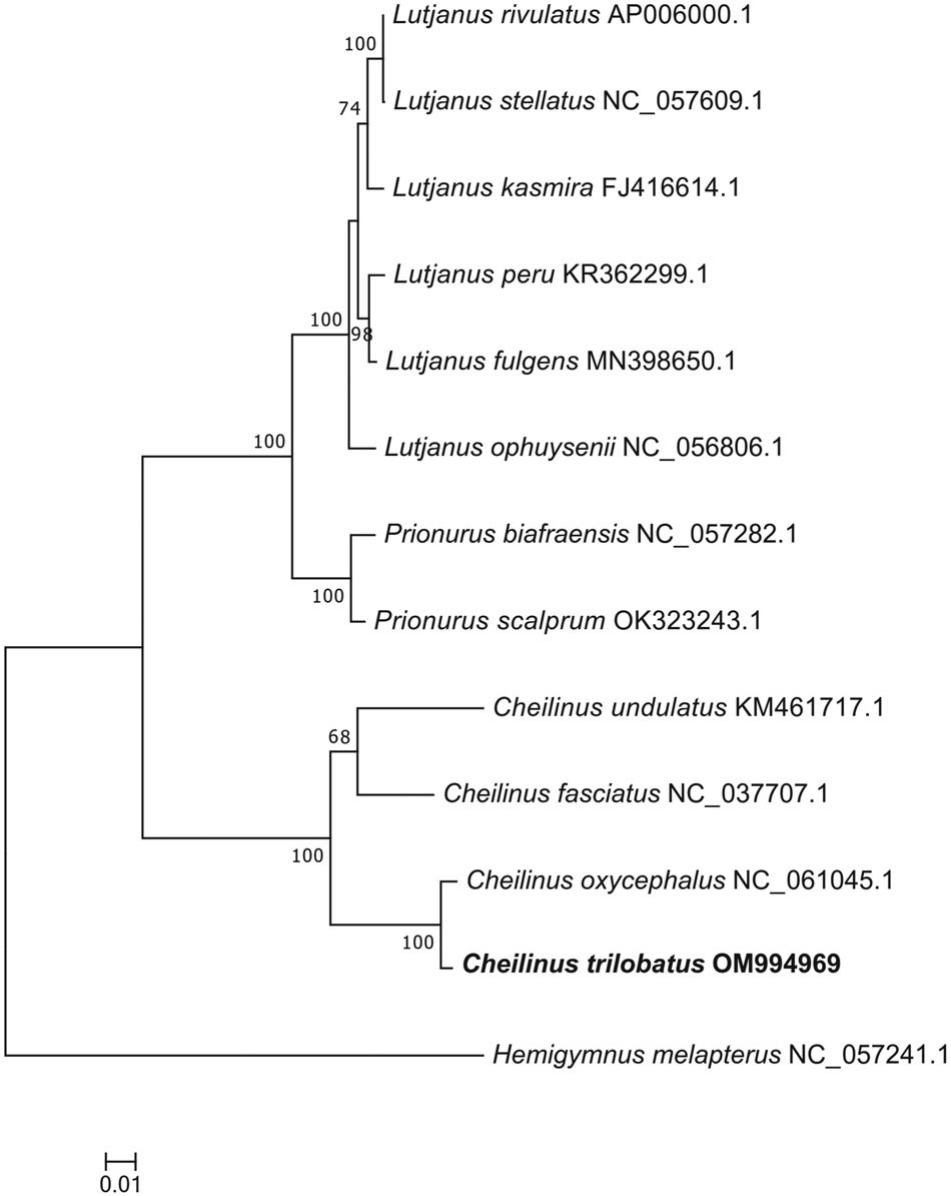
Maximum-likelihood (ML) phylogenetic tree based on complete mitogenome sequences. Numbers near the nodes represent ML bootstrap values. The following sequences were used: *Cheilinus oxycephalus* NC_061045.1, *Cheilinus fasciatus* NC_037707.1, *Cheilinus undulatus* KM461717.1, *Lutjanus stellatus* NC_057609.1, *Lutjanus rivulatus* AP006000.1, *Lutjanus kasmira* FJ416614.1, *Lutjanus fulgens* MN398650.1, *Lutjanus peru* KR362299.1, *Lutjanus ophuysenii* NC_056806.1, *Prionurus biafraensis* NC_057282.1, *Prionurus scalprum* OK323243.1, and *Hemigymnus melapterus* NC_057241.1.

To further investigate the phylogenetic relationships of *Cheilinus* and the position of *C. trilobatus*, phylogenetic trees were constructed based on the whole mitochondrial genome (Figure 3). The phylogenetic result indicated that *C. trilobatus* was closely related to *Cheilinus oxycephalus* than *C. undulatus* and *Cheilinus fasciatus*, which was congruent with the result from the barcode of life data system (Ward et al. 2009). This study provides an important molecular resource for further study on the genus *Cheilinus*.

## Ethical approval

The data collection of fishes was carried out with the permission of related institution, and complied with national or international guidelines and legislation.

## Data Availability

The genome sequence data that support the findings of this study are openly available in GenBank of NCBI at https://www.ncbi.nlm.nih.gov under the accession number OM994969. The associated BioProject, SRA, and Bio-Sample numbers are PRJNA820490, SRR18531815, and SAMN27010482, respectively.
